# Overexpression of Sarcoendoplasmic Reticulum Calcium ATPase 2a Promotes Cardiac Sympathetic Neurotransmission via Abnormal Endoplasmic Reticulum and Mitochondria Ca^2+^ Regulation

**DOI:** 10.1161/HYPERTENSIONAHA.116.08507

**Published:** 2017-03-08

**Authors:** Julia Shanks, Neil Herring, Errin Johnson, Kun Liu, Dan Li, David J. Paterson

**Affiliations:** From the Department of Physiology, Anatomy and Genetics, Burdon Sanderson Cardiac Science Centre and BHF Centre of Research Excellence, Oxford, United Kingdom (J.S., N.H., K.L., D.L., D.J.P.); and Sir William Dunn School of Pathology, Oxford, United Kingdom (E.J.).

**Keywords:** hypertension, rats, inbred SHR, sarcoplasmic reticulum calcium-transporting ATPases, stellate ganglion, sympathetic nervous system

## Abstract

Reduced cardiomyocyte excitation–contraction coupling and downregulation of the SERCA2a (sarcoendoplasmic reticulum calcium ATPase 2a) is associated with heart failure. This has led to viral transgene upregulation of SERCA2a in cardiomyocytes as a treatment. We hypothesized that SERCA2a gene therapy expressed under a similar promiscuous cytomegalovirus promoter could also affect the cardiac sympathetic neural axis and promote sympathoexcitation. Stellate neurons were isolated from 90 to 120 g male, Sprague–Dawley, Wistar Kyoto, and spontaneously hypertensive rats. Neurons were infected with Ad-mCherry or Ad-mCherry-hATP2Aa (SERCA2a). Intracellular Ca^2+^ changes were measured using fura-2AM in response to KCl, caffeine, thapsigargin, and carbonylcyanide-*p*-trifluoromethoxyphenylhydrazine to mobilize intracellular Ca^2+^ stores. The effect of SERCA2a on neurotransmitter release was measured using [^3^H]-norepinephrine overflow from 340 to 360 g Sprague–Dawley rat atria in response to right stellate ganglia stimulation. Upregulation of SERCA2a resulted in greater neurotransmitter release in response to stellate stimulation compared with control (empty: 98.7±20.5 cpm, n=7; SERCA: 186.5±28.41 cpm, n=8; *P*<0.05). In isolated Sprague–Dawley rat stellate neurons, SERCA2a overexpression facilitated greater depolarization-induced Ca^2+^ transients (empty: 0.64±0.03 au, n=57; SERCA: 0.75±0.03 au, n=68; *P*<0.05), along with increased endoplasmic reticulum and mitochondria Ca^2+^ load. Similar results were observed in Wistar Kyoto and age-matched spontaneously hypertensive rats, despite no further increase in endoplasmic reticulum load being observed in the spontaneously hypertensive rat (spontaneously hypertensive rats: empty, 0.16±0.04 au, n=18; SERCA: 0.17±0.02 au, n=25). In conclusion, SERCA2a upregulation in cardiac sympathetic neurons resulted in increased neurotransmission and increased Ca^2+^ loading into intracellular stores. Whether the increased Ca^2+^ transient and neurotransmission after SERCA2A overexpression contributes to enhanced sympathoexcitation in heart failure patients remains to be determined.

## Abstract 2

Supplemental Digital Content is available in the text.

Heart failure remains a predominant cause of mortality and morbidity globally and is characterized by a loss in efficient excitation–contraction coupling^1,2^ that leads to reduced inotropy. Downregulation of the SERCA2a (sarcoendoplasmic reticulum Ca^2+^ ATPase 2a), a key protein in cardiomyocyte excitation–contraction coupling, has been identified as a therapeutic target in both clinical^1,3^ and animal models of heart failure.^4^ Increasing myocyte SERCA2a levels by gene transfer in isolated human myocytes^5^ and preclinical animal models with heart failure^6,7^ restores cardiac inotropy and myocyte Ca^2+^ handling, without proarrhythmic side effects.^4^ Indeed, early small-scale clinical trials for the treatment of heart failure demonstrated positive results for outcome and biological safety after intracoronary injection of adeno-associated virus (AAV) type 1 SERCA2a. Prespecified clinical end points, including the 6-minute walk test, peak oxygen consumption, and left ventricular end-systolic pressure all improved.^8,9^ However, recent results from a larger phase 2 double-blind, placebo-controlled trial (CUPID2 [Calcium Upregulation by Percutaneous Administration of Gene Therapy in Cardiac Disease]) failed to meet primary clinical end points.^10,11^

Adeno viruses (Ad) and AAV are powerful tools for altering gene expression because of their high transfection efficiency and low risk of pathogenicity.^12^ They also have increased efficiency at infecting multiple cell types, including myocytes, neurons, and retinal cells,^13^ if broad-spectrum promoters are used (eg, cytomegalovirus).^10,14,15^ Therefore, it is conceivable that overexpression of AAV SERCA2a when given into the coronary circulation might also transduce the neural cardiac axis, resulting in a deleterious performance. In particular, SERCA and impairment of its regulatory protein phospholamban have been implicated in modulating depolarization-induced Ca^2+^ transients in sympathetic neurons,^16^ thus promoting neurotransmission.^17^ This neural phenotype is a well-established negative prognostic indicator in patients with heart failure.^18–21^

We therefore tested the hypothesis that enhancing SERCA2a gene expression with a cytomegalovirus promoter facilitates cardiac sympathetic neurotransmission via abnormal endoplasmic reticulum (ER) and mitochondrial intracellular Ca^2+^ handling in normal stellate neurons. Furthermore, we tested whether dysregulation of SERCA contributes to Ca^2+^ impairment in a model of cardiac sympathetic dysautonomia.

## Methods

### Animals

Age- and weight-matched male 4- to 5-week (90–120 g), Sprague–Dawley (SD, n=46), spontaneously hypertensive rat (SHR, n=22) and normotensive Wistar Kyoto (WKY, n=20) rats, in addition adult 16- to 18-week (350–380 g) male SD rats (n=20), and 9- to 10-month SHR (n=3) and WKY rats (n=3), were purchased from Envigo (Harlan, Bicester, United Kingdom) and housed under standard laboratory conditions. The investigation conformed to the Guide for the Care and Use of Laboratory Animals published by the US National Institutes of Health (NIH Publication No. 85-23, revised 1996) and the Animals (Scientific Procedures) Act 1986 (United Kingdom). Procedures were performed under British Home Office license requirements (PPL 30/3131).

### Viral Constructs

Viral constructs were manufactured commercially (Vector BioLabs, Malvern, PA). Viruses were constructed under a nonspecific cell type cytomegalovirus promoter to the same construct of human ATP2Aa as used in the CUPID trials. Ad-mCherry used was for control experiments (stock: 1×10^10^ PFU/mL), and Ad-mCherry-hATP2Aa used to up regulate SERCA2a expression (human ATP2A2a, with mCherry driven under its own cytomegalovirus promoter; stock: 1.6×10^10^ PFU/mL).

### Statistics

All statistical analysis was performed using GraphPad Prism (GraphPad Software, San Diego, CA). Data are presented as means±SEM. Analysis was performed using paired or unpaired Student *t* test as appropriate after testing and confirming all data sets were normally distributed. For all experiments, statistical significance was accepted at *P*<0.05.

An expanded materials and methods section is available in the online-only Data Supplement.

## Results

### Confirmation of SERCA2a Gene Transfer Into the Right Atria by Western Blot

**Figure 1.**
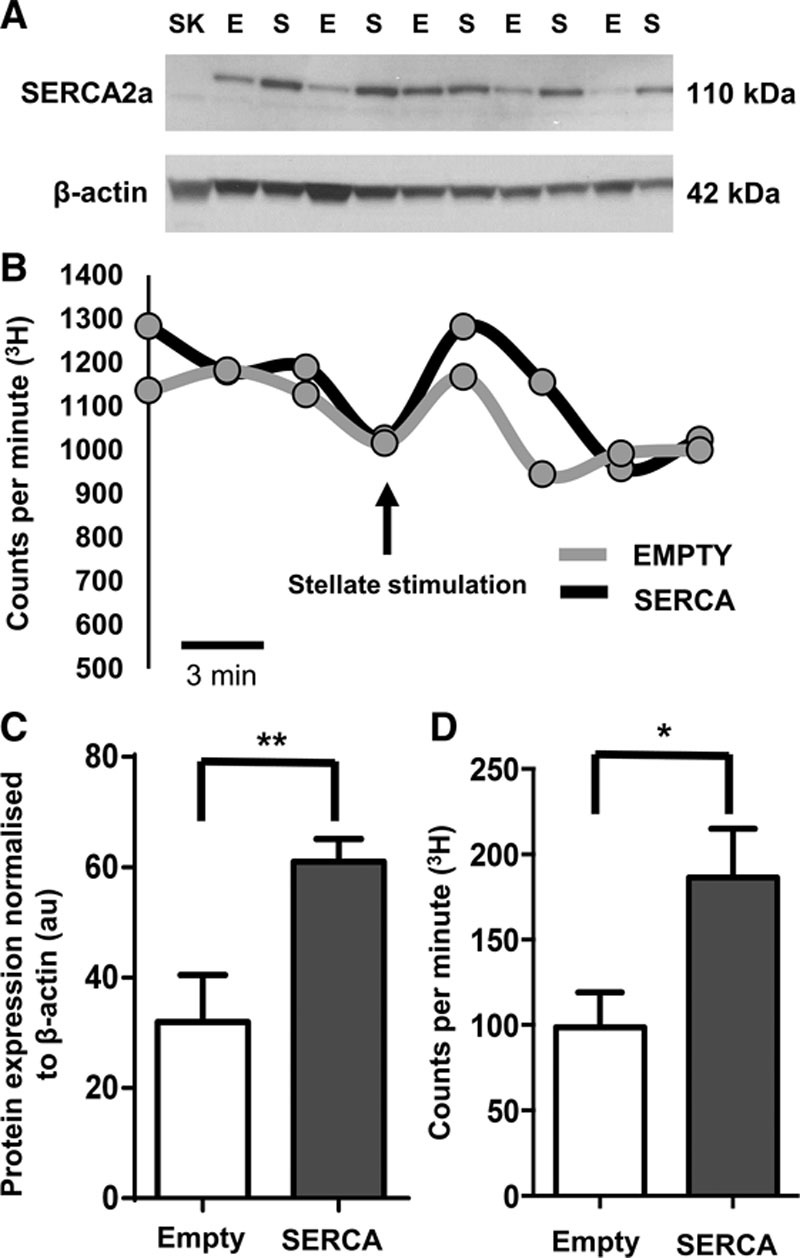
**A**, Western blot of right atrial tissue from adult (16 to 18 wk, 350–380 g) Sprague–Dawley (SD) rats who received right atrial percutaneous injection and viral gene transfer 5 d before dissection. **B**, Representative raw data traces showing [^3^H]-norepinephrine (NE) release from 350 to 380 g SD rat right atria in response to stellate stimulation (5 Hz, 1 minute), samples taken every 3 minutes, arrow indicates the time point at which the right stellate was stimulated, data point after stimulation taken as the peak in counts per minute (cpm). **C**, SERCA2a (sarcoendoplasmic reticulum calcium ATPase 2a) expression is significantly higher in atria receiving Ad-mCherry-SERCA2a gene transfer (S) than those receiving Ad-mCherry empty gene transfer (E) in which only endogenous SERCA2a is seen. No SERCA2A expression in skeletal muscle (SK) negative control, β-actin loading control expressed in all lanes. **P<0.01. **D**, Group mean data of delta CPM of [^3^H]-NE release (empty; n=7; SERCA; n=8). *P<0.05.

Percutaneous right atrial injection of SERCA2a or mCherry empty (3×10^9^ PFU/mL) was confirmed by Western blot analysis (Figure 1A). Atrial myocytes endogenously express SERCA2a, and this expression level was significantly enhanced with atrial transfection with Ad-SERCA2a, when normalized to loading control (Figure 1C) (empty: 31.9±8.5%, n=6; SERCA: 60.9±4.1%, n=7; **P<0.01).

### Effect of Right Stellate Stimulation on [^3^H]-NE Release After Gene Transfer

Right atrial injection of the SERCA2a viral vector transgene significantly increased [^3^H]-norepinephrine [^3^H]-NE) release in response to right stellate stimulation compared with atria that received injection of mCherry empty vector (Figure 1B and 1D; empty: 98.7±20.5 cpm, n=7; SERCA: 186.5±28.41 cpm, n=8; *P<0.05). This demonstrates that overexpression of SERCA2a can directly increase sympathetic neurotransmission.

### Intracellular Free Ca^2+^ Transients in Ad-SERCA2a–Transduced Stellate Neurons of the SD Rat

**Figure 2.**
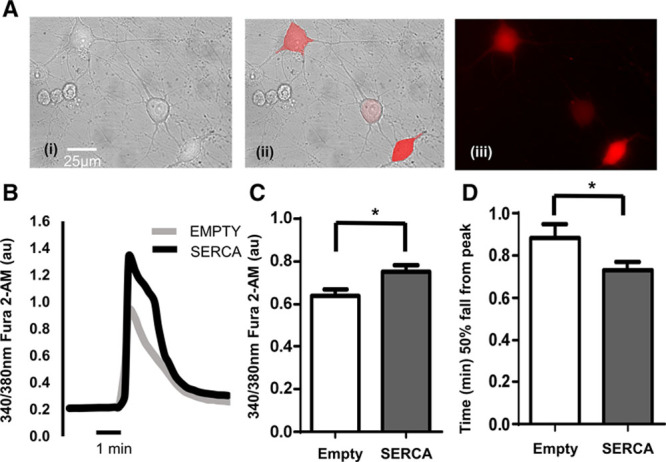
**A**, Ad-mCherry-hATP2Aa transfected stellate ganglia neurons from (4 to 5 wk, 90–120 g) Sprague–Dawley (SD) rat. (**i**) Bright field image, (**ii**) composite, and (**iii**) excitation at 587 nm to excite mCherry fluorescent tag. Only cells expressing mCherry fluorescence were used for experiments. **B**, Example raw data trace from isolated stellate ganglia neurons of the young SD rat (gray line, Ad-mCherry [empty]; black line, Ad-mCherry-hATP2Aa [SERCA (sarcoendoplasmic reticulum calcium ATPase)]) exposed to 50 mmol/L of KCl (30 s) to depolarize the neuron resulting in an increase in intracellular free Ca^2+^ ([Ca^2+^]_i_). **C**, Group mean data showing peak depolarization-evoked intracellular free Ca^2+^ increase between Ad-mCherry (gray; n=57) and Ad-mCherry-hATP2Aa (black; n=68) transfected stellate neurons. **D**, Group mean data of 50% fall time of ([Ca^2+^]_i_) from the peak (Ad-mCherry, gray; n=37; Ad-mCherry-hATP2Aa, black; n=42). *P<0.05.

Isolated stellate ganglia neurons from 4-week-old normotensive SD rats were transfected with either Ad-mCherry (empty) or Ad-mCherry-hATP2Aa (SERCA2a). Transfection of the desired gene was confirmed by only selecting cells for further experiments, which expressed the mCherry tag under 587nm excitation (Figure 2A) because gene transfer is not homogeneous. An example of the evoked intracellular free Ca^2+^ concentration change [Ca^2+^]_i_ is shown in Figure 2B with group mean data (Figure 2C). SD stellate ganglion neurons overexpressing SERCA2a exhibit a significantly greater depolarization-induced Ca^2+^ transient than those infected with the mCherry empty vector (empty: 0.64±0.03 au, n=57; SERCA: 0.75±0.03 au, n=68; *P<0.05). The time taken for the peak of the [Ca^2+^]_i_ to fall by 50% was also significantly shorter in the SERCA2a-treated neurons (empty: 0.88±0.06 s, n=37; SERCA: 0.73±0.04 s, n=68; *P<0.05; Figure 2D).

### ER Ca^2+^ Handling Within SD Stellate Neurons

**Figure 3.**
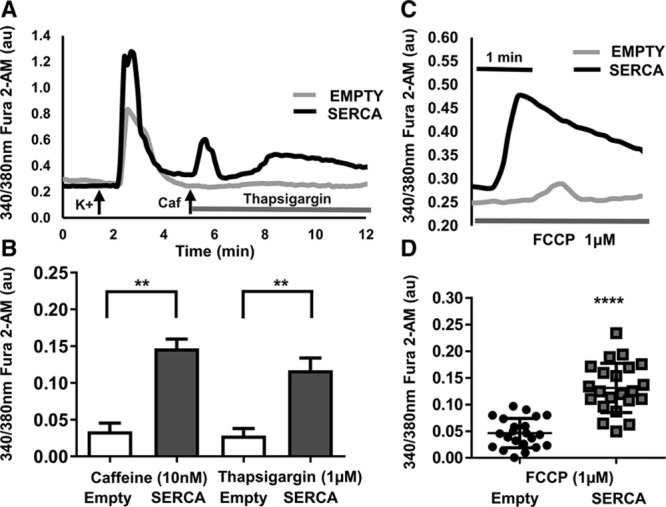
**A**, Representative raw data trace showing fluorescence ratio of fura-2AM to assess the effect of SERCA (sarco-endoplasmic reticulum calcium ATPase) gene transfection on endoplasmic reticulum (ER) Ca^2+^ handling. The effect of caffeine (10 mmol/L for 30 s at 5 min; to empty ER Ca^2+^ store) and thapsigargin (1 μmol/L thapsigargin from 5 min; to prevent ER Ca^2+^ reuptake) evoked intracellular Ca^2+^ changes in isolated Sprague–Dawley (SD) stellate neurons transfected with mCherry empty or SERCA. **B**, Group mean data of [Ca^2+^]i in response to caffeine (empty: n=35; SERCA: n=45) and thapsigargin (empty: n=33; SERCA: n=42). **P<0.01. **C**, Representative raw data trace showing the effect mCherry empty or SERCA2a gene transfection had on mitochondrial Ca^2+^ uncoupling by carbonylcyanide-*p*-trifluoromethoxyphenylhydrazine (FCCP; 1 μmol/L) in isolated SD rat stellate ganglia neurons. **D**, Group mean data of [Ca^2+^]_i_ in response to FCCP (empty: n=22; SERCA: n=22). ****P<0.0001.

Ca^2+^ concentrations from the ER were measured by monitoring [Ca^2+^]_i_ change in response to caffeine (10 mmol/L for 30 seconds) to deplete ER Ca^2+^ stores and thapsigargin (1 μmol/L) to block ER Ca^2+^ reuptake. SERCA2a-treated cells had a significantly greater increases in [Ca^2+^]_i_ in response to caffeine (Figure 3A and 3B; empty: 0.03±0.01 au, n=35; SERCA: 0.15±0.01 au, n=45) and thapsigargin (empty: 0.03±0.001 au, n=33; SERCA: 0.12±0.01 au, n=42; **P<0.01). This would support the idea that the increased depolarization-induced Ca^2+^ transients observed in the SERCA2a-treated neurons are likely because of greater SERCA2a expression, resulting in greater Ca^2+^ load in the ER which is in turn mobilized by calcium-induced calcium release. Not all neurons in wells incubated with the virus expressed the mCherry tag (efficiency ≈ 60–70%). In some experiments within one field of view, separate neurons with varying expression levels could be seen. Within dishes infected with the SERCA2a transgene, cells not expressing mCherry had caffeine and thapsigargin responses similar to empty vector–treated neurons.

### Effect on Mitochondrial Ca^2+^ Handling Within SD Stellate Neurons

The effect of SERCA2a overexpression on mitochondrial Ca^2+^ handling was observed by using the proton uncoupler carbonylcyanide-*p*-trifluoromethoxyphenylhydrazine (FCCP; 1 μmol/L) that causes depolarization of the mitochondrial membrane. This results in depletion of Ca^2+^ stores and inhibition of further mitochondrial Ca^2+^ uptake.^16,22^ Application of FCCP produced a transient increase in [Ca^2+^]_i_ (Figure 3C). This change was significantly higher in the SERCA2a-transduced neurons compared with empty treated cells (Figure 3D; empty: 0.05±0.005 au, n=22; SERCA: 0.13±0.009 au, n=22; **P<0.01). This indicates that not only is ER Ca^2+^ loading increased by upregulating SERCA2a expression but that the concentration of whole cell bound intracellular Ca^2+^ had also increased.

### Intracellular Free Ca^2+^ Transients in Ad-SERCA2a–Transduced Stellate Neurons of the SHR and WKY

SHR have previously been shown to exhibit high sympathetic drive, even before the onset of hypertension,^16,17,23^ and develop heart failure with increasing age,^24^ compared with the normotensive WKY. Therefore, the effect of SERCA2a upregulation was studied in these neurons to better reflect the disease model.

**Figure 4.**
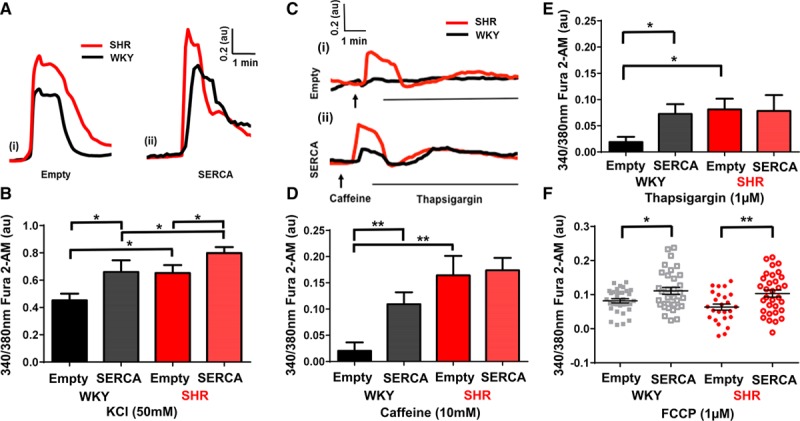
**A**, An example raw data traces showing free intracellular Ca^2+^ change in response to KCl (30 s; 50 mmol/L) depolarization of isolated stellate ganglia neurons from Wistar Kyoto (WKY; black) and spontaneously hypertensive rats (SHR; red), transfected with mCherry empty (i) or SERCA (sarco-endoplasmic reticulum calcium ATPase; ii). **B**, Group mean data of peak depolarization-induced free intracellular Ca^2+^ change (WKY: empty; n=17; SERCA; n=13; SHR: empty; n=18; SERCA; n=25). *P<0.05. **C**, Representative raw data trace showing the effect of SERCA gene transfection on endoplasmic reticulum (ER) Ca^2+^ handling, caffeine (10 mmol/L) and thapsigargin (1 μmol/L), in isolated WKY (black) and SHR (red) stellate neurons transfected with mCherry empty (i) or SERCA (ii). Group mean data of [Ca^2+^]_i_ in response to caffeine (**D**) and thapsigargin (**E**; WKY: empty; n=17; SERCA; n=13; SHR: empty; n=18.;SERCA; n=25). **F**, Effect on mitochondrial Ca^2+^ uncoupling by carbonylcyanide-*p*-trifluoromethoxyphenylhydrazine (FCCP; 1 μmol/L) in isolated WKY and SHR rat stellate ganglia neurons with either mCherry empty or SERCA gene transfection. Group mean data of WKY: empty; n=32; SERCA; n=33; And SHR: empty; n=25; SERCA; n=32. **P<0.01.

Representative raw data traces (Figure 4A) illustrate that [Ca^2+^]_i_ transients were significantly greater in neurons of the SHR compared with the WKY rat (as previously reported^17^) in both experimental conditions, when the 2 cell types were carrying either the (i) empty or (ii) SERCA2a transgene (*P<0.05). Moreover, in concordance with the results seen in the SD stellate neurons, SERCA2a overexpression increased [Ca^2+^]_i_ transients compared with empty control cells in both the WKY and the SHR, group mean data (Figure 4B; WKY, empty: 0.45±0.05 au, n=17; SERCA: 0.66±0.09 au, n=13; SHR, empty: 0.65±0.06 au, n=18; SERCA: 0.80±0.04 au, n=25; *P<0.05).

### Transmission Electron Microscopy of ER of Young 4-Week SHR and WKY

**Figure 5.**
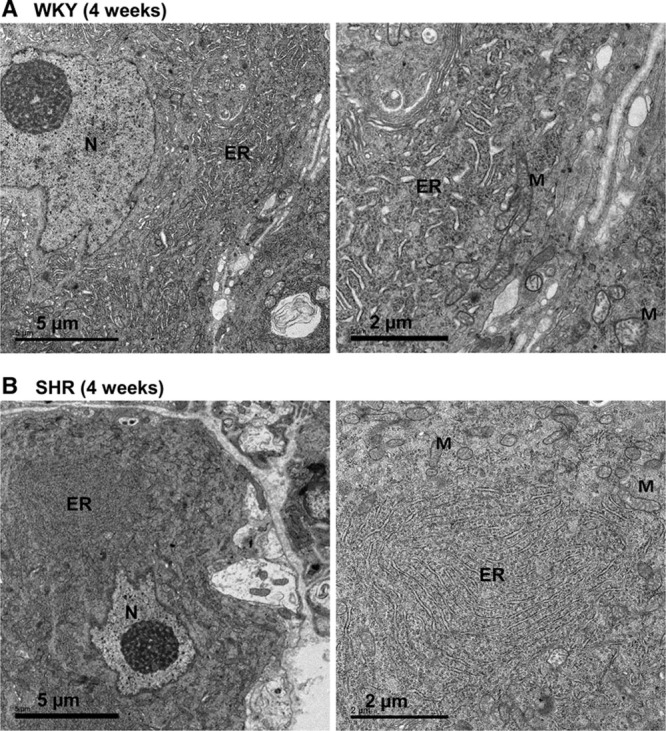
Transmission electron micrographs of stellate neurons in young Wistar Kyoto (WKY) rat (**A**) and spontaneously hypertensive rats (SHR; **B**). Endoplasmic reticulum (ER) morphology is affected in stellate neurons from SHRs. WKY rat shows a dispersed ER network, whereas SHR rats exhibit regions of highly enriched sheet-like ER. M indicates mitochondria; and N, nucleus.

Interestingly, transmission electron microscopy images of the ER from stellate ganglia of 4-week-old SHR and WKY rats shows a striking difference in ER structure and organization (Figure 5). In SHR rats, the ER is organized into spatially compact sheets compared with the more disperse and varied ER form observed in WKY (Figure 5), suggesting that structural changes might underpin ER Ca^2+^ handling differences observed in the SHR compared with the WKY.

### ER Ca^2+^ Handling in Prehypertensive SHR and Age-Matched WKY Stellate Neurons

SERCA2a-treated cells of WKY neurons showed increased [Ca^2+^]_i_ in response to mobilization of ER load with caffeine (WKY; empty: 0.02±0.02 au, n=17; SERCA: 0.11±0.02 au, n=13; Figure 4C and 4D) and thapsigargin (empty: 0.02±0.01 au, n=17; SERCA: 0.07±0.02 au, n=13; *P<0.05; **P<0.01; Figure 4E). Although mCherry empty-treated SHR neurons had a greater increase in [Ca^2+^]_i_ in response to caffeine and thapsigargin compared with mCherry empty-treated WKY neurons, no further increase was observed after treatment with SERCA2a (SHR caffeine, empty: 0.16±0.04 au, n=18; SERCA: 0.17±0.02 au, n=25; thapsigargin, empty: 0.08±0.02 au, n=18; SERCA: 0.08±0.03 au, n=25; Figure 4C–4E). These data suggest that stellate neurons of the prehypertensive SHR present altered ER Ca^2+^ handling compared with normotensive WKY controls, even before upregulation of the SERCA2a transporter, in keeping with the observation that there is more ER per unit of cell volume in SHR neurons. Moreover, under basal conditions, upregulation of SERCA2a seems to have no further compounding effect on ER loading in the SHR, suggesting that the ER may already be working at full Ca^2+^ load.

### Effect of Mitochondrial Ca^2+^ Handling in SHR and Age-Matched WKY Stellate Neurons

In SHR stellate neurons, transfection with SERCA2a was still able to increase the mitochondrial Ca^2+^ store as measured using the mitochondrial membrane uncoupler FCCP (Figure 4F), despite not increasing ER Ca^2+^ load. This may explain why SERCA2a transfection still leads to an increased overall depolarization-induced [Ca^2+^]_i_ transient in these neurons. WKY neurons overexpressing SERCA2a also had a greater increase in [Ca^2+^]_i_ in response to FCCP compared with mCherry empty controls (Figure 4F; WKY, empty: 0.08±0.006 au, n=32; SERCA: 0.11±0.01 au, n=33; SHR, empty: 0.06±0.009 au, n=25; SERCA: 0.10±0.01 au, n=32; **P<0.01).

## Discussion

The key findings of this study are as follows: (1) The SERCA2a protein (predominantly thought to be the cardiomyocyte isoform of SERCA^1,2^) can be transduced into rat stellate neurons. (2) Upregulating SERCA2a in normal rat stellate neurons leads to greater depolarization-induced Ca^2+^ transients, as well as greater ER and mitochondrial Ca^2+^ load. (3) Right atrial percutaneous injection of the SERCA2a virus results in increased [^3^H]-NE release in response to right stellate stimulation. (4) Stellate neurons from SHRs have a greater ER calcium load than the WKY and have a greater abundance of ER per unit cell volume compared with WKY neurons. (5) SERCA2a overexpression does not increase ER load further in SHR neurons, but still increases the depolarization-induced Ca^2+^ transient, potentially through increased mitochondrial calcium loading.

### SERCA2a Upregulation Results in Enhanced Intracellular Ca^2+^ Handling in Sympathetic Neurons

The predominant neuronal SERCA isoforms are SERCA3 in the cerebral cortex,^25^ SERCA2b in hippocampal pyramidal neurons,^26^ with low levels of SERCA2a expression in superior cervical ganglia neurons of young SHR and WKY rats, with no observed differences between the 2 strains.^16^ Incorporation of the SERCA2a isoform into cardiac stellate neurons resulted in increased depolarization-induced Ca^2+^ transients in normotensive and prehypertensive animal models and increased ER Ca^2+^ load within stellate neurons isolated from young SD and WKY rats. Increased depolarization-induced Ca^2+^ transients have previously been described in sympathetic neurons of the neonatal to adult SHR compared with age-matched WKY rat controls.^17^ Isolated neurons from the superior cervical ganglia have alluded to increased ER Ca^2+^ load in young prehypertensive SHR,^16^ analogous to the data shown here in stellate neurons exposed to the mCherry empty viral vector. Young prehypertensive SHR had larger ER Ca^2+^ stores and greater caffeine-evoked ER Ca^2+^ release compared with WKY neurons. This may be related to the activity of the SERCA transporter that is under the control of regulatory protein phospholamban. Phospholamban (PLN) is a small phosphoprotein that can regulate the activity of the SERCA. Dephosphorylated PLN is an inhibitor of SERCA, whereas phosphorylation of PLN relieves its inhibition.^27^ We have previously reported that expression of phosphorylated (Ser16) compared with total PLN is reduced in prehypertensive SHR superior cervical ganglion neurons. Therefore, less dephosphorylated PLN may increase SERCA activity, resulting in more rapid reuptake of calcium into the ER and faster recovery of the intracellular calcium transient in the prehypertensive SHR.^16^ We have now evaluated SERCA2a and PLN expression in stellate ganglia of WKYs and SHRs at 9 to 10 months of age when the SHR develops impaired left ventricular function.^28^ At this age, these preliminary data suggest there is no apparent statistical difference in the expression of both SERCA2a (WKY: 1.000±0.0003 au, n=3; SHR: 0.572±0.232 au, n=3) and PLN (WKY: 1.01±0.01 au, n=3; SHR: 1.52±0.52 au, n=3). However, given the small sample size and the difficulty in extracting sufficient levels of protein from this small ganglion, we cannot rule out that physiological reductions in SERCA2a occurred.

Although SERCA2a overexpression increased ER Ca^2+^ load in WKY stellate neurons to a level comparable with the SHR, no difference was seen in the ER load of the SHR between SERCA2a overexpression and control. This indicates that part of the faulty and heightened Ca^2+^ handling observed in stellate neurons of the SHR may be because of already maximal Ca^2+^ loading of the ER. Electron microscopy of the young WKY and prehypertensive SHR indicates that the ER is more densely and structurally organized within stellate neurons of the SHR. Although ER load was not altered in SHR neurons with SERCA2a overexpression, an increase in depolarization-induced Ca^2+^ transients in the SHR was still observed that may be because of both a greater ER and a mitochondrial Ca^2+^ load and release after subsequent depolarization. It remains to be seen whether sympathetic neuronal ER Ca^2+^ is already maximally loaded in a heart failure model, and whether upregulating SERCA2a in these neurons would also increase the depolarization-induced calcium transient and subsequent NE release. However, the fact that there is no difference in the expression of SERCA2a or PLN in 9- to 10-month-old SHR and WKYs and SERCA2a overexpression is still able to increase the depolarization-induced Ca^2+^ transient in prehypertensive SHRs when the ER is fully loaded makes potentiation of sympathetic neurotransmission likely.

Mitochondria are fundamentally important for maintaining cellular Ca^2+^ homeostasis, as well as energy production. Mitochondrial research has shown them to be necessary for regulating Ca^2+^ in many physiological processes, including vasomotion in blood vessels^29^ and accumulation of Ca^2+^ when cytosolic levels are low in synaptosomes.^30^ Functional or direct coupling of the ER and mitochondria has been suggested in many cell types, including sympathetic neurons,^31,32^and mitochondria have been indicated to be involved in the uptake of ER-released Ca^2+^, regulating neuronal excitability.^32^ FCCP depolarization within this study has been used as a means to assess mitochondrial Ca^2+^ load, although it does not rule out that part of the Ca^2+^ transient observed with FCCP could be because of a coupling between the mitochondria and the ER. The difference observed with FCCP-liberated free Ca^2+^ in SHR SERCA2a neurons indicates that FCCP is predominantly releasing Ca^2+^ from a non-ER store. The transient time scales of the application of FCCP reduce the chance that the observed Ca^2+^ transients are because of changes in energy production of the cell inhibiting SERCA activity by reducing ATP production.

Increased mitochondrial Ca^2+^ concentrations alter the mitochondrial membrane potential, with elevated mitochondrial Ca^2+^ levels being linked to impaired mitochondrial energetics^33^ and increased oxidative stress within cardiomyocytes.^34,35^ Previously, SERCA2a transgene in cardiomyocytes has been predicted to be protective at preventing mitochondrial stress by ensuring resting intracellular Ca^2+^ levels remain low,^36^ thereby protecting the myocardial energetics of the cell.^37,38^ We could not directly record the mitochondrial membrane potential in this study because of the mCherry fluorescent tag exhibiting cross fluorescent specter with tetramethylrhodamine ethyl ester (used to measure mitochondrial membrane potential^16^). Therefore, it remains to be established whether SERCA overexpression protects neuronal energetics.

### SERCA2a Overexpression Results in Increased Neurotransmitter Release in Response to Right Stellate Stimulation

Right atrial injection of adeno- and lentiviral constructs has previously been established as viable tools to upregulate target genes of interest that can modulate neurotransmission in cardiac autonomic nerves.^39–43^ We established whether the increased depolarization-induced Ca^2+^ transients and elevated intracellular Ca^2+^ handling observed in isolated stellate neurons functionally translates. Direct stimulation of the isolated stellate ganglia infected with the SERCA2a gene construct resulted in significantly greater neurotransmitter release from the atria and potentially greater postsynaptic excitability. Although we have highlighted the effect that incorporation of atrial-injected SERCA2a gene transfer on sympathetic neurons may have, we cannot rule out that the viral construct could also be expressed in both cardiac afferent and vagal nerve fibers. This may alter local network processing and subsequent NE release, for example, via the release of other neurotransmitters and neuropeptides.

### Limitations

As has previously been described,^39^ transfection rate with adenovirus is not 100% efficient (≈70%); therefore, it was vital that before Ca^2+^ imaging experiments, only cells expressing the target gene were used. Our mCherry fluorescent tag confirmed cells had integrated the transgene and were expressing the protein of interest. Inefficient transfection rate without a method of monitoring gene delivery could result in inconsistent or false-negative results.

Although within this study we have highlighted the effects of incorporation of SERCA2a into cardiac sympathetic nerves, the stellate ganglia contain a heterogeneous profile of sympathetic efferent cardiac and noncardiac neurons.^44,45^ The promiscuous nature of the cytomegalovirus-Ad viruses used means it is highly likely that all neurons of the stellate ganglia had the potential to overexpress SERCA2a after viral transfection. Overexpression of SERCA2a could have resulted in altered intracellular Ca^2+^ handling in neurons innervating noncardiac, as well as cardiac, tissue. Cardiac neurons and noncardiac neurons from the rat stellate ganglia do not have clearly distinct morphologies or resting membrane potentials^45^ and have been identified through their electrophysiological responses to cardiac nerve stimulation^45^ or through their endogenous activity in relation to the cardiac cycle,^44^ which cannot be assessed in isolated cultured neurons during calcium imaging. The inclusion of some noncardiac neurons may have introduced variability within our experimental groups although all sympathetic neurons responded similarly with SERCA2a overexpression.

To assess the functional significance of SERCA2a gene therapy coinfecting the cardiac autonomic axis, in vivo large mammal models would have to be studied to establish whether this gene transfer approach translated into more cardiac excitability. Specifically, this would need to be performed in an established heart failure model. Because adenovirus and AAV have high specificity of transfection for both myocytes and nerve cells,^13^ it is plausible that the intracoronary perfusion of SERCA2a gene constructs within the CUPID trials could have resulted in gene transfection into cardiac sympathetic neurons, as well as cardiomyocytes.^9^ Studies in spinal cord injury have shown that AAV–green fluorescent protein transduction close to the site of injury can result in the spread of a green fluorescent protein–tagged fluorescence throughout the spinal cord and into the central nervous system.^46^ This suggests the need for cell specific targeting in gene therapy.

### Perspectives

Overexpression of SERCA2a using a promiscuous cytomegalovirus viral promoter resulted in increased neurotransmission and altered intracellular Ca^2+^ handling within neurons isolated from the stellate ganglia of normotensive rats. Recent use of gene therapy in clinical trials of heart failure failed to show a beneficial effect of SERCA2a overexpression targeted at myocytes, constructed under a similar promiscuous promoter. The potential for off target expression of the SERCA2a transgene in other cell types, including sympathetic neurons, may have compounded these results. Whether SERCA2a overexpression has a similar effect on the cardiac sympathetic neural axis in heart failure remains to be established.

## Acknowledgments

Transmission electron microscopy work was undertaken in the Dunn School Electron Microscopy Facility, and we are grateful to Anna Pielach for preparing the samples for transmission electron microscopy analysis.

## Sources of Funding

This work was supported by the British Heart Foundation (BHF) Centre of Research Excellence, Oxford. N.H. is a British Heart Foundation Intermediate Fellow (FS/15/8/3115).

## Disclosures

None.

## Supplementary Material

## Novelty and Significance

### What Is New?

- The first study to demonstrate that upregulating a myocyte isoform of SERCA (sarcoendoplasmic reticulum calcium ATPase) into isolated sympathetic neurons affects intracellular Ca^2+^ handling dynamics.

### What Is Relevant?

- Dysregulation of SERCA2a in heart failure has led to the development of therapeutic strategies to upregulate its activity in myocytes. It is feasibly possible these viruses may also infect sympathetic neurons altering their Ca^2+^ handling phenotypes.
- An observed increase in endoplasmic reticulum (ER) Ca^2+^ capacity within the prehypertensive spontaneously hypertensive rats may contribute to the sympathetic hyperactivity.

### Summary

An adenoviral gene transfer technique to upregulate SERCA2a in isolated sympathetic neurons resulted in altered intracellular Ca^2+^ handling, greater ER Ca^2+^ load, and greater depolarization-induced Ca^2+^ transients within 4- to 5-week Sprague–Dawley and Wistar Kyoto rats. Upregulation of SERCA2a expression had no effect on the ER Ca^2+^ load within 4- to 5-week prehypertensive spontaneously hypertensive rats, and transmission electron microscopy revealed a more disperse and varied ER appearance compared with the Wistar Kyoto, indicating that increased ER Ca^2+^ capacity and altered intracellular Ca^2+^ handling within the prehypertensive spontaneously hypertensive rats may contribute to the observed sympathetic hyperactivity. Therapeutic targets aimed at reducing rather than increasing ER load within sympathetic neurons in hypertension may be beneficial.
