# Supplementary material for: Overexpression of Sarcoendoplasmic Reticulum Calcium ATPase 2a Promotes Cardiac Sympathetic Neurotransmission via Abnormal Endoplasmic Reticulum and Mitochondria Ca2+ Regulation
Source: Hypertension. 2017 Mar 8;69(4):625–32. doi: 10.1161/HYPERTENSIONAHA.116.08507 (PMC5344179; doi:10.1161/HYPERTENSIONAHA.116.08507)
Supplement: Supplementary file 1 [file hyp-69-625-s001.docx]

**Online methods supplement**

**Overexpression of SERCA2a promotes cardiac sympathetic neurotransmission via abnormal endoplasmic reticulum and mitochondria Ca^2+^ regulation.**

Julia Shanks^1^, Neil Herring^1^, Errin Johnson^2^, Kun Liu^1^, Dan Li^1^, David J. Paterson^1^

^1^Burdon Sanderson Cardiac Science Centre and BHF Centre of Research Excellence,

Department of Physiology, Anatomy and Genetics, Sherrington Building, Oxford, OX1 3PT, UK

^2^Sir William Dunn School of Pathology, South Parks Road, Oxford, OX1 3RE, UK

Julia Shanks, MSc, DPhil

Neil Herring, DPhil, MRCP, FHRS

Errin Johnson, PhD

Kun Liu, MD

Dan Li, MD, DPhil

David J. Paterson, DPhil, D.Sc

**Correspondence:**

David J. Paterson D.Phil, D.Sc

Burdon Sanderson Cardiac Science Centre

Department of Physiology, Anatomy and Genetics,

Sherrington Building, Parks Road,

Oxford, OX1 3PT, UK

**Animals**

SD rats were used as a proof of principle study in a normotensive model. We also assessed the effect that upregulating SERCA2a expression has on Ca2+ handling in stellate neurons from young SHR. These animals express cardiac sympathetic hyperactivity compared to age matched WKY controls prior to the development of hypertension[1](#ENREF_17), and progress to develop heart failure with age[2](#ENREF_22). Cardiac sympathetic hyperactivity is a negative prognostic indictor in the development of hypertension and heart failure in clinical and animal models. The majority of experiments and all cellular imaging were performed in young animals (90-120 g). This allowed for maximal cell yield and maximum success rate of cell culture, whilst still using animal models that displayed sympathetic phenotypes concordant with their adult counterparts. Older rats (350-380g) were used for the percutaneous viral gene transfer experiments to better reflect the anatomical adult model.

**Isolation and preparation of sympathetic post-ganglionic neurons**

Sympathetic neurons were isolated from the stellate ganglion that predominantly innervate the heart[3](#ENREF_23), from 90g-120 g rats as previously described[4](#ENREF_24). Rats were rendered unconscious using general anesthesia (3% isoflurane and 97% oxygen) then humanely killed by an approved Home Office schedule 1 method: cervical dislocation followed by exsanguination. Dissociated neurons were plated onto poly-D-lysine/ laminin substrate coated cover slips (5mm), placed at 37oC 5% CO_2_ for 3 days before use. The medium used for the isolation and culture of the isolated postganglionic sympathetic neurons were based on modifications of those previously described[25](#ENREF_25). In brief, dissected and cleaned ganglion were placed in a 10ml falcon tube containing collagenase type 4 (1 mg/ml in Ca2+ and Mg+2-free phosphate-buffered saline, PBS) for 14 min at 37 oC in a shaking water bath. The collagenase was removed and replaced with trypsin TRL (2mg/ml in PBS) for 14 min 37oC. The neurons were then washed for 2 x 3 min washes in an L-15 based blocking medium, followed by 2 x 3 min washes in L-15 based plating medium to stop the action of the digestion enzymes.

Ganglia were titrated into a single-cell suspension with a narrow opening fire polished Pasteur pipette 5 times. After the trituration the solution was left to rest for 1.5 minutes to allow any large tissue fragments to settle to the base of tube whilst leaving the single cells in suspension. The top 0.5 ml of solution was then removed and placed in a plastic petri dish (2.5 mm diameter). 0.5 ml of fresh L-15 based plating medium was added to the falcon tube and this process was repeated 5 times. The cell suspension solution was then added to wells containing Poly-D lysine and laminin coated cover slips (6 mm), and incubated at 37oC 5% CO_2_ for 3 days before use.

**Measurement of free intracellular Ca2+ concentration**

*Virus infection*

Isolated stellate ganglion neurons were incubated with the virus, either Ad-mCherry (2x107 PFU/ml), or Ad-SERCA2a-mCherry (6.4x107 PFU/ml) for 24 hours post isolation. Cells were then changed to fresh virus free media for 48 hours prior to imaging. Cells were checked for mCherry fluorescence to confirm expression of the viral gene transferred protein before each experiment.

*Protocol*

Neurons were loaded with 2.5 μM Fura-2/AM for 40 min at room temperature in Tyrodes solution, then washed for 10min with room temperature Tyrodes solution to allow the intracellular Fura-2/AM to de-esterify. Loaded neurons were transferred to a temperature controlled (37 oC) gravity fed perfusion chamber (volume 500 μl); flow rate 3 ml/min, visualised on a Nikon Eclipse TE200-U microscope with a 40X oil immersion lens. Cells expressing fluorescence of the mCherry tag (excitation 587 nm; emission 610 nm) were selected for study.

Images were acquired every 3 seconds using a photometrics CoolSNAP HQ2 camera. Neurons were excited alternately at 340 nm and 380 nm and detecting emission at 510 nm. The ratio of 380/340 nm gave a measure of free intracellular Ca2+ concentration change.

Application of KCl (50 mM; 30 s), caffeine (10 mM; 30 s), thapsigargin (1 μM; 10 min), and FCCP (1 μM; 3 min), were used to evoked transient intracellular Ca2+ changes.

**Measurement of local 3H-norepinephrine release from isolated double atria.**

350-380 g SD rats were given a percutaneous right atrial injection (under 3% isoflurane) of either Ad-mCherry or Ad-SERCA2a-mCherry (3x109 PFU/ml) in 300 μl PBS. Experiments were performed 5-6 days post gene transfer.

Spontaneously beating right atria with intact sympathetic innervations and right stellate ganglion were isolated from 350-380 g SD rats, and transferred to a pre-heated (37±0.2oC), water jacketed, carbogen-aerated water bath containing 3 ml Tyrode solution. The atria were pinned flat over a silver stimulating electrode, and the right stellate ganglion was threaded over a two prong stimulating electrode and fixed in place. The method for determining local 3H-norepinephrine (NE) release was based on one previously described[26](#ENREF_26). Briefly, following a 20min equilibration period, the double atria preparation was incubated with 5 µM 3H-NE (0.185 MBq, Perkin Elmer) and ascorbic acid (30 µM, Sigma). The atria were field stimulated at 5Hz (15 V, 1 ms pulse width) for 10 s every 30 s, for 30 min to facilitate uptake of 3H-NE into the transmitter stores of the pre-synaptic terminal. Following 3H-NE incubation excess radioactivity was washed from the preparation with Tyrode’s solution superfusion for 45 min, at a rate of 3 ml/min. Bath solution was then replaced every 3 min for 51 min. A 0.5 ml sample from each solution change was added to 4.5 ml scintillation liquid (Ecoscint A, National Diagnostics) and the amount of radioactivity measured (counts per minute, CPM) using a liquid scintillation counter (Tri-Carb 2800TR, Perkin Elmers Life Science). At 16 min the atria were field stimulated at 5 Hz for 1 min, at 41 min the right stellate were stimulated at 5 Hz for 1 min. The 3H-NE outflow from the right atria was presented as change in counts per minute in response to stimulation of the stellate compared to the data point taken immediately before the peak. Post experiment right atria were snap frozen to be used in western blot and confirm transgene expression.

**Western blot**

Right atria from 350-380 g SD rats post 3H-NE overflow measurements were immediately snap frozen in liquid nitrogen. Atria were lysed in the presence of proteases inhibitors to prevent target protein breakdown. Sample protein concentrations were quantified according to the Bradford protein assay. 20 μg of total protein was separated by SDS-PAGE. The expression of SERCA2a (abcam 1:1000) and β-actin (Sigma 1:1000) were detected. Immunodetection was based on chemiluminescence quantification (Western Lightning Plus, Perkin Elmer Life Science). The results were normalized to β-actin that served as a loading control, and antibody specificity tested using rat thigh skeletal muscle lysate as a negative control to SERCA2a.

**Electron microscopy**

Excised ganglion tissue was immediately immersed in pre-warmed fixative (2.5% glutaraldehyde, 2% PFA in 0.1 M sodium cacodylate buffer, pH 7.2) for approximately 2 h at room temperature, then stored at 4 °C for two weeks before further processing. Samples were then washed on a rotor in 0.1 M sodium cacodylate buffer pH 7.2 for 4x 30 min, then in 100 mM glycine in the same buffer for 60 min and finally for 2x 30 min in sodium cacodylate buffer. Samples were then incubated in 1% osmium tetroxide in the same buffer for 2 h at 4°C, washed with water for 3x 20 min and incubated in 0.5% uranyl acetate (aqueous) overnight at 4°C. Samples were washed briefly with water, then taken through a graded ethanol series (30%, 50%, 70%, 80%, 90% and 95% ethanol for 15 min each, then 100% ethanol for 2 h, with 3 solution changes during this time). Samples were gradually infiltrated with TAAB TLV epoxy resin, starting with 25% resin for 1 hr, 50% resin for 2 h, 75% resin for 1.5 h and 100% resin overnight. Samples were transferred to a fresh 2ml tube of resin, spun for ~30s in a mini centrifuge then placed on a rotator at room temperature for several hours. This step was repeated four more times, with another overnight incubation in between.  Individual tissue pieces were transferred to Beem capsules filled with fresh resin, then polymerised for 48 hrs at 60 °C. Blocks were sectioned using a Leica UC7 ultramicrotome with a diamond knife (Diatome). Ultrathin sections (90nm) were placed on 200 mesh Copper grids, post-stained with Reynold’s lead citrate for 5 min, and imaged on a FEI Tecnai 12 Transmission Electron Microscope (TEM)EM operated at 120 kV using a Gatan OneView CMOS camera.

**Solutions and Drugs**

Tyrode solution for isolated postganglionic sympathetic neuron experiments contained (in mmol/L: NaCl 145, KCl 5, HEPES 10, glucose 10, CaCl_2_ 2 and MgCl_2_ 1) (pH 7.38-7.42, 37±0.5oC). All drugs were stored as stock solutions and made to the desired concentration on the day. All drugs underwent no more than one freeze thaw cycle. Tyrode solution for isolated right atria/right stellate ganglia experiments contained (mmol/L NaCl 120, KCl 4.7, MgSO_4_ 1.2, NaHCO_3_ 25, CaCl_2_ 2, KH_2_PO_4_ 1.2 and glucose 11) and was aerated with 95 % O_2_/5 % CO_2_ (pH 7.4).

1. Shanks J, Manou-Stathopoulou S, Lu CJ, Li D, Paterson DJ, Herring N. Cardiac sympathetic dysfunction in the prehypertensive spontaneously hypertensive rat. *American Journal of Physiology Heart and Circulatory Physiology* 2013;305:H980-986.

2. Conrad CH, Brooks WW, Hayes JA, Sen S, Robinson KG, Bing OH. Myocardial fibrosis and stiffness with hypertrophy and heart failure in the spontaneously hypertensive rat. *Circulation* 1995;91:161-170.

3. Pardini BJ, Lund DD, Schmid PG. Innervation patterns of the middle cervical--stellate ganglion complex in the rat. *Neuroscience Letters* 1990;117:300-306.

4. Wang L, Li D, Plested CP, Dawson TA, Teschemacher AG, Paterson DJ. Noradrenergic neuron-specific overexpression of nNOS in cardiac sympathetic nerves decreases neurotransmission. *Journal of Molecular and Cellular Cardiology* 2006;41:364-370.
